# TCMIP v2.0 Powers the Identification of Chemical Constituents Available in Xinglou Chengqi Decoction and the Exploration of Pharmacological Mechanisms Acting on Stroke Complicated With Tanre Fushi Syndrome

**DOI:** 10.3389/fphar.2021.598200

**Published:** 2021-07-15

**Authors:** Ping Wang, Shuang Wang, Hong Chen, Xiaofang Deng, Luoqi Zhang, Haiyu Xu, Hongjun Yang

**Affiliations:** ^1^ Institute of Chinese Materia Medica, China Academy of Chinese Medical Sciences, Beijing, China; ^2^ College of Pharmacy, Heilongjiang University of Chinese Medicine, Harbin, China; ^3^ College of Traditional Chinese Medicine, Shenyang Pharmaceutical University, Shenyang, China; ^4^ Shaanxi Institute of International Trade and Commerce, Xianyang, China; ^5^ Experimental Research Center, China Academy of Chinese Medical Sciences, Beijing, China

**Keywords:** TCMIP, integrative pharmacology strategy, UPLC-QTOF-MS/MS, Xinglou Chengqi decoction, Tanre Fushi syndrome

## Abstract

Xinglou Chengqi (XLCQ) decoction, composed of three botanical drugs and one inorganic drug, is used in clinics during the treatment of acute stroke complicated with Tanre Fushi (TRFS) syndrome in China. However, its active ingredients and the molecular mechanism have not been clarified. So, we aimed to preliminarily characterize its chemical constituents and investigate its pharmacological mechanisms using an integrative pharmacology strategy, including component analysis, network prediction, and experimental verification. We employed UPLC-QTOF-MS/MS to describe the chemical profile of XLCQ, Integrative Pharmacology-based Network Computational Research Platform of Traditional Chinese Medicine (TCMIP v2.0, http://www.tcmip.cn/), to assist in identifying the chemical components and predict the putative molecular mechanism against acute stroke complicated with TRFS, and LPS-stimulated BV-2 cells to verify the anti-neuroinflammatory effects of luteolin, apigenin, and chrysoeriol. Altogether, 197 chemical compounds were identified or tentatively characterized in the water extraction of XLCQ, 22 of them were selected as the key active constituents that may improve the pathological state by regulating 27 corresponding targets that are mainly involved in inflammation/immune-related pathways, and furthermore, luteolin, apigenin, and chrysoeriol exhibited good anti-neuroinflammatory effects from both protein and mRNA levels. In summary, it is the first time to employ an integrative pharmacology strategy to delineate 22 constituents that may improve the pathological state of stroke with TRFS by regulating 27 corresponding targets, which may offer a highly efficient way to mine the scientific connotation of traditional Chinese medicine prescriptions. This study might be a supplement for the deficiency of the basic research of XLCQ.

## Introduction

Stroke, characterized by a high incidence rate, high mortality, and a high disability rate, is a devastating cerebrovascular event that occurs as a result of cerebral vascular occlusion (ischemic stroke) or burst/bleeding (hemorrhagic stroke), leading to cerebral blood flow disruption, physical disability, and multiple functional impairment, which seriously threatens human health and quality of life (Feigin et al., 2016). At present, the main treatment to ischemic stroke is early thrombolysis to restore blood flow and achieve vascular recanalization, but most patients cannot get thrombolytic therapy due to the limitation of treatment time window or other contraindications. Therefore, it is particularly urgent to find safe and effective drugs for stroke prevention and treatment with a clear mechanism.

Traditional Chinese medicine (TCM), characterized by lower side effects, is often thought to be an alternative pharmacotherapy for prevention and rehabilitation intervention of ischemic stroke in China (Liu et al., 2018). As early as in 1981, Academician Yongyan Wang observed that about 74.2% of stroke patients were accompanied by “Tanre Fushi” (TRFS) syndrome (Wang, 1981) that manifests as abdominal distension, constipation, bad breath, and dry throat, and then he developed the “Huatan Tongfu” treatment strategy in the following year (Wang et al., 1982). In 1986, he created “Huatan Tongfu” decoction, which was later renamed as “Xinglou Chengqi” (XLCQ) decoction, to treat acute stroke in clinics. At the same time, the indication and opportunity for the correct application of XLCQ decoction were given (Wang et al., 1986). XLCQ decoction is composed of three botanical drugs and one inorganic drug, namely, *Trichosanthes kirilowii* Maxim. (Gualou, GL) 30–40 g, *Arisaema erubescens* (Wall.) *Schott* (Dannanxing, DNX) 6–10 g, *Rheum palmatum L.* (DaHuang, DH) 10–15 g, and *Natrii sulfas* (Mangxiao, MX) 10–15 g. The main component of *Natrii sulfas* is Na_2_SO_4_•10H_2_O, which belongs to a mineral medicine.

Accumulating clinical practices have proven that the effective power of the Western medicine treatment for acute stroke could be enhanced obviously when combined with XLCQ, especially when the patient is suffering with TRFS (Chen et al., 2017; Zhen, 2017; Liu and Lei, 2019; Wang et al., 2020a; Han, 2020; Jiang et al., 2020; Lu and Wang, 2020; Yao et al., 2020). Although XLCQ has almost been used for 40 years in clinics, its chemical composition has not been systematically characterized, and its pharmacological mechanism is limited to anti-inflammatory (Du et al., 2009; Wand, 2016; Zhen, 2017; Zhao et al., 2018), anti–free radical injury (Wu and Jiang, 2005; Zhou et al., 2016), inhibition of neuronal damage (Liu et al., 2012a; Yu et al., 2018), and anti-neuronal apoptosis (Liu et al., 2012b).

**FIGURE 1:**
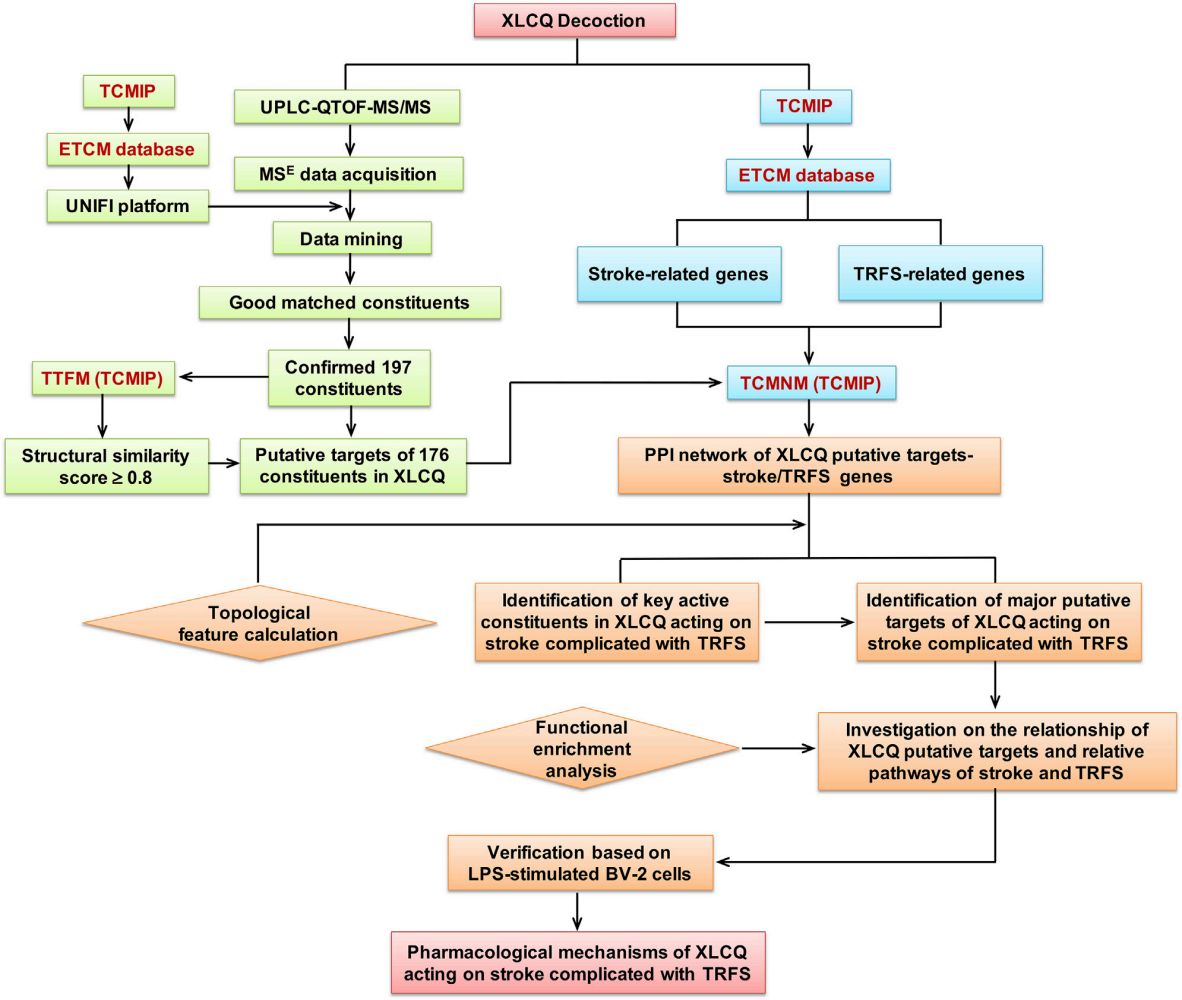
The scheme of the present study.

Therefore, in the present study, we aimed to describe the chemical profiles and explore the underlying pharmacological mechanisms of XLCQ acting on stroke with TRFS through the following scheme, as shown in Figure 1: 1) analyzing XLCQ chemical components by UPLC-QTOF-MS/MS, 2) collecting the information of chemical components from Integrative Pharmacology-based Network Computational Research Platform of Traditional Chinese Medicine (TCMIP v2.0, http://www.tcmip.cn/), 3) predicting the putative targets of the identified components and collecting the genes of stroke and TRFS from TCMIP, 4) constructing a “component targets-stroke/TRFS genes” network to select the candidate targets and the main active components of XLCQ, 5) functional enrichment analysis for investigating the underlying molecular mechanisms of XLCQ acting on stroke complicated with TRFS, and 6) verifying the anti-neuroinflammatory effects of luteolin, apigenin, and chrysoeriol based on LPS-simulated BV-2 cells.

## Materials and Methods

### Chemicals and Reagents

HPLC-grade acetonitrile and methanol were purchased from Merck KGaA (Darmstadt, Germany); HPLC-grade formic acid from Sigma-Aldrich (St. Louis, MO, United States); chloroform (100068), isopropanol (80109218), and anhydrous alcohol (10009218) from Sinopharm (Beijing, China); and DEPC-treated water (sc-204391) from Santa Cruz Biotechnology (CA, United States). Deionized water (18.2 MΩ cm) was purified by a Milli-Q system (Millipore Co., Billerica, United States). *Trichosanthes kirilowii* Maxim. (place of production: Hebei Province; batch number: 190112010), *Arisaema erubescens* (Wall.) Schott (place of production: Sichuan Province; batch number: D180903001), *Rheum palmatum L.* (place of production: Sichuan Province; batch number: 18111201), and *Natrii Sulfas* (place of production: Sichuan Province; batch number: DD0181) were purchased from Shengshi Baicao Pharmaceutical Co., Ltd. and authenticated by Mrs. Xirong He, a research assistant of China Academy of Chinese Medical Sciences (Beijing). The voucher specimens were deposited in the Institute of Chinese Materia Medica, China Academy of Chinese Medical Sciences.

Fetal bovine serum (FBS,10270–106), penicillin–streptomycin (10,000 U/mL, 15140122), and 0.05% trypsin-EDTA (1X, 25300–054) from Gibco BRL Co. (Boise, Idaho, United States); Dulbecco’s Modified Eagle’s Medium-high glucose (DMEM/High Glucose, SH30022.01) from HyClone Laboratories (Logan, UT, United States); dry powder of phosphate-buffered saline (PBS, P1010), dimethyl sulfoxide (DMSO, D8371), and sterile deionized water (F0025) from Solarbio Life Sciences (Beijing, China); lipopolysaccharide (LPS, 0111:B4, L2630) from Sigma-Aldrich Crop. (St. Louis, MO, United States).

Luteolin (BT1191), apigenin (BT443), and chrysoeriol (BT2582) were borrowed from Beijing Beite Renkang Biomedical Technology Co., Ltd. (Beijing, China); IL-1β (CSB-E08054m) and TNF-α (CSB-E04741) ELISA kits from Cusabio (Beijing, China); CCK-8 kit from Dojindo (Kumamoto ken, Japan); and TB Green® Premix Ex Taq™ (RR420A) from TAKARA BIO INC. (Chiryu Shi, Japan).

### Preparation of Herbal Extracts

The prescription is composed of GL, DNX, DH, and MX with a dose proportion of 15:3:5:5. GL and DNX were soaked in 10 volumes of water for 60 min before boiling for 30 min. Then DH was added and boiling was maintained for another 30 min. MX was dissolved in the decoction that was filtered by eight layers of gauze. The residue was boiled with 8 volumes of water for another 30 min, and the decoction was filtered as before. The two combined filtrates were freeze-dried (Lab-1D-80E; Beijing boyaikang Experimental Instrument Co., Ltd., Beijing, China) at −80°C with a paste rate at 26.96%, and the powder was pressed through a 60-mesh sieve. The fine powder was dissolved into 10 volumes of 70% MeOH and extracted with an ultrasonic wave for 15 min before 1-μl aliquots were transferred to autosampler vials for analysis.

### Instrumentation and UPLC-QTOF-MS/MS Conditions

The analysis was performed on a Waters Acquity UPLC I-Class system (Waters Corp., Milford, United States) equipped with a binary pump, an online vacuum degasser, an autosampler, and an automatic thermostatic column oven, coupled with a Waters Xevo G2-S Q-TOF Mass System (Manchester, United Kingdom) equipped with electrospray ionization (ESI). The data were recorded by Masslynx V4.1 (Waters Corporation, Milford, United States). Unifi software (Waters Corporation, Milford, United States) was used for peak detection and compound preliminary identification.

Chromatographic separation was performed on a Waters Acquity UPLC HSS T3 column (100 mm × 2.1 mm, i.d., 1.8 μm) maintained at 35 °C, and a linear gradient of (A) 0.1% (v/v) formic acid in deionized water and (B) acetonitrile containing 0.1% (v/v) formic acid was used for the elution procedure, as follows: 0–3 min, 0.2–2% B; 3–5 min, 2–5% B; 5–6 min, 5–8% B; 6–10 min, 8–16% B; 10–10.2 min, 16–19% B; 10.2–14.5 min, 19–25% B; 14.5–15 min, 25–30% B; 15–15.5 min, 30–40% B; 15.5–18 min, 40–50% B; 18–20 min, 50–70% B; 20–21 min, 70–98% B; 21–24 min, 98% B; 24–24.1 min, 98–0.2% B; and 24.1–26 min, 0.2% B. The flow rate was set at 0.5 ml/min, and a 1-μl aliquot was set as the injection volume.

The QTOF-MS data were collected in a full scan auto mode from 0 to 26 min in both positive and negative ion modes. Based on the best response for most of the compounds, the optimal parameters were set as follows: mass range, 50–1,500 Da; source temperature, 100 °C; desolvation temperature, 450°C; desolvation gas flow, 900 l/h; sampling cone, 40 V; ESI^−^ capillary voltage, 2.5 KV; and ESI^+^ capillary voltage, 0.5 KV. At low CE scan, the auto MS collision energy was 6 eV. At high CE scan, the collision energy was 30–50 eV ramp for ESI^+^ and 80–100 eV ramp for ESI^−^.

Leucine enkephalin was employed as lock mass at a concentration of 200 pg/ml in acetonitrile (0.1% formic acid): H_2_O (0.1% formic acid) (50:50, v/v) for the positive ion mode ([M + H]^+^ = 556.2771) and negative ion mode ([M−H]^−^ = 554.2615) *via* a lock spray interface.

### Data Processing

UPLC-QTOF-MS/MS system was controlled by the Masslynx 4.1 platform. The MS^E^ data collected in a continuum mode were processed using the apex peak detection and alignment algorithms in UNIFI 1.8, which enabled quasi-molecular ion peaks, salt adduct ions, and dehydration fragment ions to be analyzed as a single entity. The analysis process includes data acquisition, data mining, library searching, and report generation.

The information of chemical components GL, DNX, and DH including molecular name, molecular formulas, molecular weights, and chemical structures (mol. format) was collected from ETCM (Xu et al., 2019) (http://www.nrc.ac.cn:9090/ETCM/), as a customized library listed in Supplementary Excel S1, to assist the chemical identification, which were transferred to the UNIFI form later (Supplementary Excel S2). The additive ions of [M + H]^+^, [M + K]^+^, [M + Na]^+^, [2M + H]^+^, and [M-e]^+^ were selected in a positive ion mode and [M + COOH]^-^, [M-H]^-^, and [2M-H]^-^ in a negative ion mode. A margin of error up to 5 mDa/10 ppm for identified components was allowed, and the matched components would be generated as predicted fragments from the structure. For unmatched components, the functional module of elemental composition and mass fragment could further assist the chemical identification. Based on the isotopic abundance, the elemental composition calculator could provide a number of possible formulas for an accurate mass peak. Mass fragment could provide fragment structures which assist the chemical identification. The final list of possible formulas could provide relative confidence denoted by an i-FIT score and 0.8 as the threshold value.

### Prediction of the Putative Targets of Chemical Constituents Available in XLCQ

The mol. formats of identified compounds were uploaded to the customer center of TCMIP to predict the putative targets using TCM target prediction and function analysis module (TTFM) according to the chemical structure similarity comparison with known drugs on the market. In order to improve the prediction accuracy, we set the structural similarity score at 0.80 (moderate∼high similarity) to select the constitute–putative target pairs (Mao et al., 2019).

### Network Construction of XLCQ Putative Target-Stroke/TRFS Gene

To investigate the relationship of XLCQ putative targets and stroke/TRFS genes, we collected a list of stroke-related genes and TRFS-related genes from disease-related gene database of TCMIP. The key words of stroke were “stroke, ischemic stroke, brain injury, cerebral ischemia, and cerebral hemorrhage.” TRFS syndrome was a combination of Yang Ming Fushi syndrome, phlegm heat obstructing lung syndrome, and wind-phlegm syndrome. So, the symptomatic phenotypes of three syndromes were used as the key words to collect the related genes, including “fever, delirium, abdominal pain, dysphoric mood, hyperhidrosis, constipation, wheezing, cough, vertigo, vomiting, abnormality of salivation, syncope, facial paralysis, and hemiplegia.”

An interaction network of XLCQ putative target–stroke/TRFS-related gene was constructed based on the links among the three gene sets using the TCM Association Network Mining Module (TCMNM) of TCMIP, which directly exhibits the major hub network according to three topological features of each node gene, including “degree,” “betweenness,” and “closeness.” Generally, the node that has the degree value 2-fold the median, and betweenness and closeness value 1-fold the median is selected as the major hub.

### Network Visualization and Functional Enrichment Analysis

To better exhibit the common targets among XLCQ, stroke, and TRFS, the Venn diagram was performed using the “Calculate and draw custom Venn diagrams” website (http://bioinformatics.psb.ugent.be/webtools/Venn/). To better exhibit the interactions of the major hubs, the network visualization was performed using CytoScape V3.8.0. To elucidate the biological functions of XLCQ putative targets, the functional enrichment analysis was performed using the database for annotation, visualization, and integrated discovery (DAVID) v6.8 (https://david.ncifcrf.gov).

### Cell Viability Evaluation

Murine BV-2 microglia cells were obtained from the Institute of Materia Medica-Chinese Academy of Medical Science (Beijing, China) and cultured in DMEM supplemented with 10% heat-inactivated FBS and antibiotics (100 U/ml streptomycin and 100 U/ml penicillin) at 37°C in an incubator [Sanyo MCO-18AIC (UV), Osaka, Japan] with a humidified atmosphere containing 5% CO_2_ and 95% O_2_. BV-2 cells were plated in 96-well plates with density at 1 × 10^4^ cells/well and incubated in the abovementioned conditions for 24 h. Luteolin, apigenin, and chrysoeriol solutions were added to different wells with final concentrations at 80, 40, 20, 10, and 5 μM and incubated for another 24 h. Cell viability was evaluated by a CCK-8 kit, and the absorbance was determined at 450 nm 2 h later by a ThermoFisher Scientific Multiskan FC Microplate Reader (MA, United States).

### Drug Treatment

BV-2 cells were plated in 24-well plates with the density at 1.5 × 10^5^ cells/well and incubated for 24 h. Luteolin, apigenin, and chrysoeriol solutions were added with final concentrations at 10 μM and incubated for 1 h. Then LPS was added to induce neuroinflammation with a final concentration of 1 μg/ml, and incubated for another 24 h. Finally, the supernatant was collected by centrifugation at 12,000 r and 4 °C for 10 min, which was used for IL-1β and TNF-α analyses according to the instructions of ELISA kits.

### Real-Time Reverse Transcription–Polymerase Chain Reaction (RT-qPCR)

The total RNA was isolated using TRNzol Universal Reagent (TIANGEN, DP424, China), and 1 μg RNA was reverse-transcribed to cDNA using FastKing gDNA Dispelling RT SuperMix (TIANGEN, KR118-02, China) by Veriti 96-Well Thermal Cycler PCR (Thermo Fisher, 4375786, United States) according to the manufacturer’s instructions. A single-stranded cDNA was amplified by PCR with primers for IL-1β, TNF-α, PIK3CA, AKT1, NFKB1, NFKB2, CREB1, HSP0AA1, and β-actin, whose primer sequences are shown in Table 1. PCR was performed using a real-time fluorescence quantitative PCR instrument (Roche, LightCycler480 II, Germany) by the following two-step PCR amplification procedure: 1 cycle of pre-degradation at 95°C for 30 s, 40 cycles of denaturation at 95°C for 5 s (ramp rate: 4.4°C/s), and then, annealing and extension at 60°C for 30 s (ramp rate: 2.2°C/s, acquisition mode: single). β-actin was selected as an internal control to evaluate the expression of the eight genes. Primers were purchased from Beijing Qingke Biotechnology Co., Ltd. (Beijing, China).

**TABLE 1:** Primers used in this study.

Primer name	Nucleotide sequence (5′-3′)	Product size
IL-1β forward	GCA​ACT​GTT​CCT​GAA​CTC​AAC​T	89bp
IL-1β reverse	ATC​TTT​TGG​GGT​CCG​TCA​ACT	
TNF-α forward	CCC​TCA​CAC​TCA​CAA​ACC​AC	133bp
TNF-α reverse	ACA​AGG​TAC​AAC​CCA​TCG​GC	
PIK3CA forward	TAT​GTC​TAC​CCT​CCA​AAT​GTC​G	128bp
PIK3CA reverse	TAC​TTC​TGC​TTG​TCG​TTG​TTT​G	
AKT1 forward	ATG​AAC​GAC​GTA​GCC​ATT​GTG	116bp
AKT1 reverse	TTG​TAG​CCA​ATA​AAG​GTG​CCA​T	
NF-κB1 forward	CAA​AGA​CAA​AGA​GGA​AGT​GCA​A	203bp
NF-κB1 reverse	GAT​GGA​ATG​TAA​TCC​CAC​CGT​A	
NF-κB2 forward	CAA​GGA​CAT​GAC​TGC​TCA​ATT​T	92bp
NF-κB2 reverse	GCC​TCT​GAA​GTT​TCT​GGA​TCA​T	
CREB1 forward	AGC​AGC​TCA​TGC​AAC​ATC​ATC	152bp
CREB1 reverse	AGT​CCT​TAC​AGG​AAG​ACT​GAA​CT	
HSP90AA1 forward	TGT​TGC​GGT​ACT​ACA​CAT​CTG​C	116bp
HSP90AA1 reverse	GTC​CTT​GGT​CTC​ACC​TGT​GAT​A	
β-actin forward	GGC​TGT​ATT​CCC​CTC​CAT​CG	154bp
β-actin reverse	CCA​GTT​GGT​AAC​AAT​GCC​ATG​T	

## Results

### Characterization and Identification of Chemical Constituents Available in XLCQ

**FIGURE 2:**
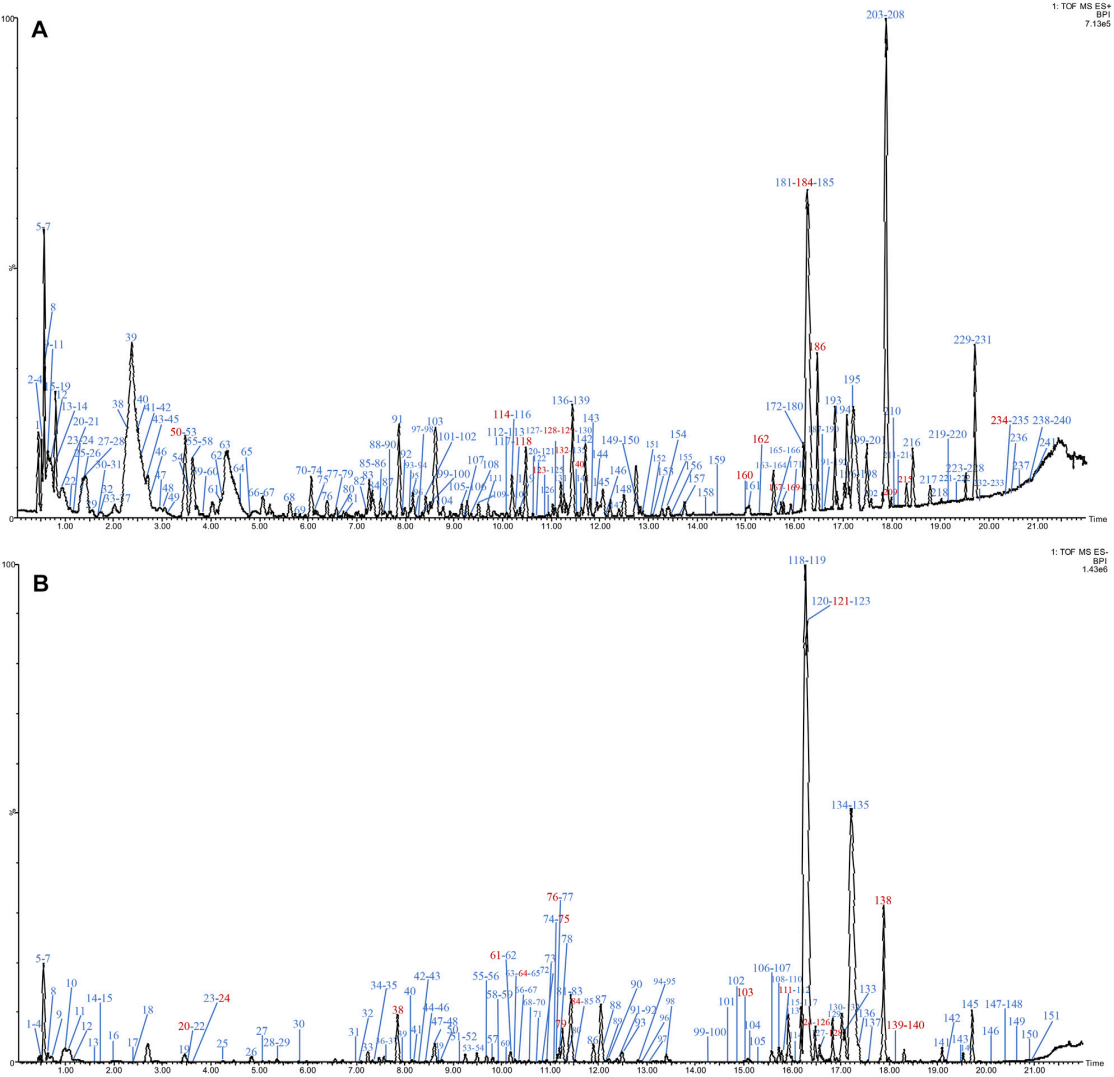
BPI chromatograms of water extracts of XLCQ **(A)**. ESI^+^ and **(B)**. ESI^−^.

**FIGURE 3:**
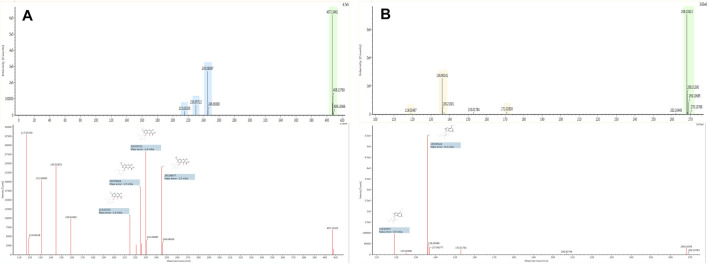
Spectrum information of torachrysone-8-O-β-D-glucoside **(A)** and arginine **(B)** automatically provided by UNIFI^TM^.

The base peak intensity (BPI) chromatograms of water extraction of XLCQ corresponding to the positive and negative ion modes are shown in Figure 2. The MS^E^ raw data obtained by UPLC-QTOF-MS/MS were processed using the UNIFI 1.8 software automatically to quickly identify the constituents by matching the detailed information with the customized library (Supplementary Excel S2). Altogether, a total of 197 compounds (152 in ESI^+^ and 116 in ESI^−^) were identified or tentatively characterized, of which 56 originated from GL, 63 from DNX, and 78 from DH. The detailed information of chemical compounds is listed in Supplementary Tables S1, S2, containing RT, M/Z, error, response, adducts, formula, name, fragments, category, and origination. The identified constituents, especially the isomers, were verified by the characteristic fragments reported in the literatures. Taking two compounds as examples, the secondary fragment information was exhibited in detail. The ion at RT = 15.56 and [M-H]^-^ = 407.1334 was primarily identified as torachrysone-8-O-β-D-glucoside (C_20_H_24_O_9_) in ESI^−^ after searching in the scientific database of UNIFI collected from ETCM, and the main fragments were m/z 245.0600 [M-H-Glc]^-^, 230.0560 [M-H-Glc-CH3]^-^, 215.0324 [M-H-Glc-2CH_3_]^−^, 159.0431 [M-H-Glc-2CH_3_-2CO]^−^, and 141.0483 [M-H-Glc-2CH_3_-3CO]^−^ (Figure 3A), which were consistent with the literature report (Gao, 2012). In the same way, ion at RT = 3.46 and [M + H]^+^ = 268.1046 was primarily identified as arginine (C_10_H_13_N_5_O_4_) in ESI^+^, and the main fragments were m/z 136.0624 and 119.0349 (Figure 3B), corresponding to loss -C_5_H_7_O_4_ and -NH_3_ in turn, which were consistent with the literature report (Wang and Han, 2018).

### Putative Targets of Chemical Constituents Available in XLCQ

**FIGURE 4:**
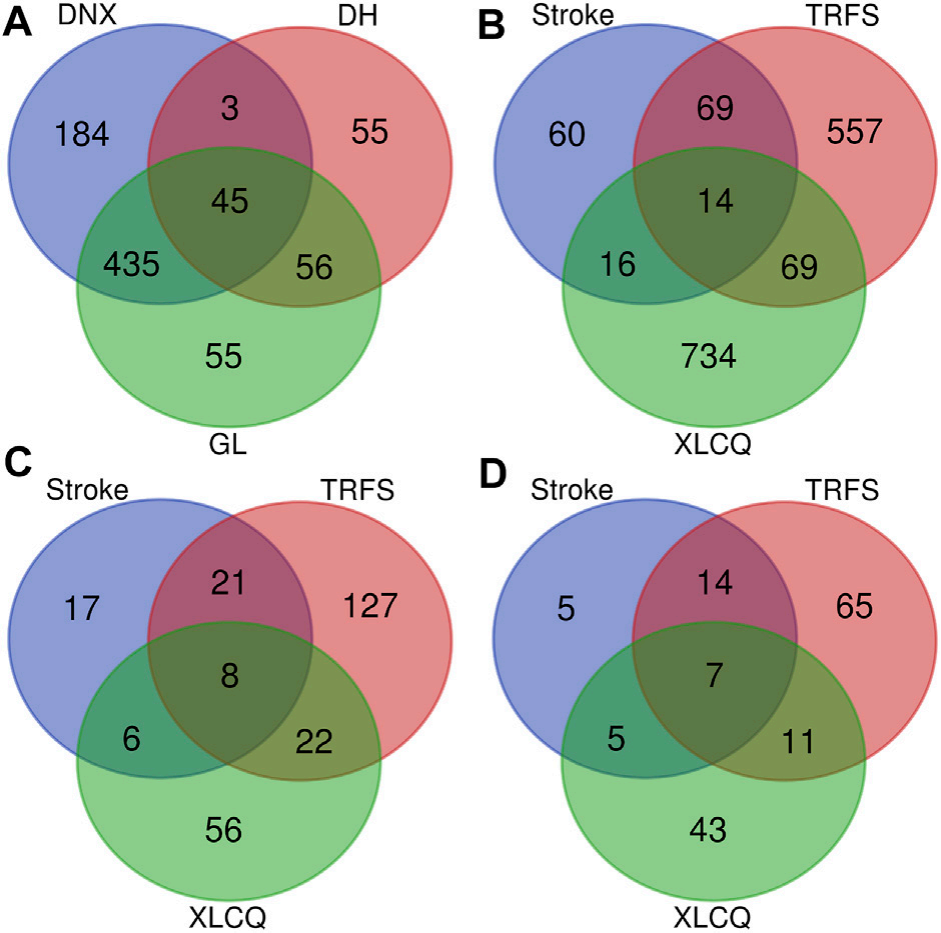
The Venn diagram of putative targets of the three herbs available XLCQ **(A)**; the Venn diagram of XLCQ putative targets, known stroke/TRFS-related genes **(B)**; the Venn diagram of 257 major hubs **(C)**; and the Venn diagram of 150 key hubs **(D)**. [GL: *Trichosanthes kirilowii Maxim.*; DNX: *Arisaema erubescens (Wall.) Schott*; DH: *Rheum palmatum L.*].

Altogether, a total of 833 putative targets were predicated based on the chemical structures of 197 primarily identified compounds using the TCM Target Prediction and Function Analysis Module of TCMIP (Supplementary Excel S3). Only 176 compounds (52 from GL, 51 from DNX, and 73 from DH) had putative targets when the Tanimoto score was set at 0.8 (moderate∼high similarity). The putative targets of GL, DNX, and DH were 591, 667, and 159, respectively. Interestingly, the three herbs had a number of common putative targets according to the prediction, indicating the potential drug–drug interactions through their common targets (Figure 4A).

### The Gene Set of Stroke and TRFS Syndrome

A total of 159 stroke-related genes and 709 TRFS-related genes were collected from the disease-related gene database of TCMIP (Supplementary Excel S3). The distribution of the 1519 genes was exhibited in the Venn diagram (Figure 4B), with XLCQ 734, stroke 159, and TRFS 709. Whereas “stroke” was a disease, “TRFS” was a syndrome, and there were still 83 common targets, indicating they may have the possible effects targets. XLCQ had 30 common targets with that of stroke and 83 with that of TRFS, indicating the possible direct targets of XLCQ acting on stroke complicated with TRFS. The common targets of XLCQ with TRFS were more than those of XLCQ with stroke, indicating that the therapeutic effect of XLCQ on TRFS may be stronger than that of stroke.

### Underlying Mechanisms of XLCQ Acting on Stroke Complicated With TRFS Syndrome

To illustrate the underlying mechanisms of XLCQ acting on stroke complicated with TRFS, an interaction network of drug target genes and disease/syndrome-related genes was constructed based on the interactions among three gene sets using the TCM Association Network Mining Module of TCMIP, and the network topological features were calculated automatically by TCMIP. Altogether, 257 hubs were selected, and detailed information is provided in Supplementary Excel S3. The target distribution of the 257 genes was exhibited in the Venn diagram (Figure 4C). The target number of XLCQ, stroke, and TRFS was 92, 52, and 178, respectively, with 30 common targets between XLCQ and TRFS, 14 between XLCQ and stroke, and 29 between stroke and TRFS.

To improve the prediction accuracy, 150 major hubs whose degree value ≥12 were selected from the 257 hubs (Supplementary Excel S3, marked in red). Among them, 31 nodes were stroke-related genes, 97 were TRFS-related genes, and 66 were XLCQ putative targets (Figure 4D).

**FIGURE 5:**
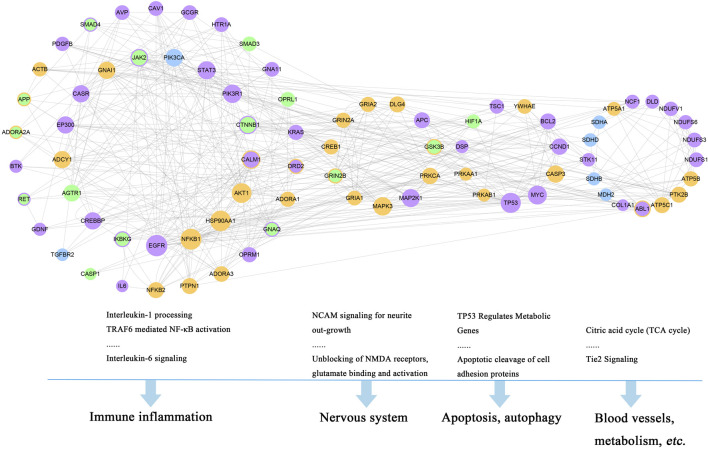
Four functional modules involved in 82 major hubs and the corresponding pathological events during the progression of stroke complicated with TRFS. (Orange nodes refer to putative targets of XLCQ; green nodes refer to known therapeutic targets of stroke; purple nodes refer to known therapeutic targets of TRFS; blue nodes refer to the common genes of three sets; and the color-overlay nodes refer to common genes of two sets.)

The functional enrichment analysis of the 150 genes was investigated by DAVID v6.8. Altogether, 85 pathways were obtained based on the Reactome Pathway Database. Among them, 52 pathways (Supplementary Excel S3, marked in bold) containing 82 genes were involved in the corresponding pathological events during the progression of stroke and TRFS, which were divided into four functional modules, including immune inflammation module, nervous system module, apoptosis, and autophagy module (Figure 5).

### Selection of Key Active Constituents of XLCQ Acting on Stroke Complicated With TRFS Syndrome

**FIGURE 6:**
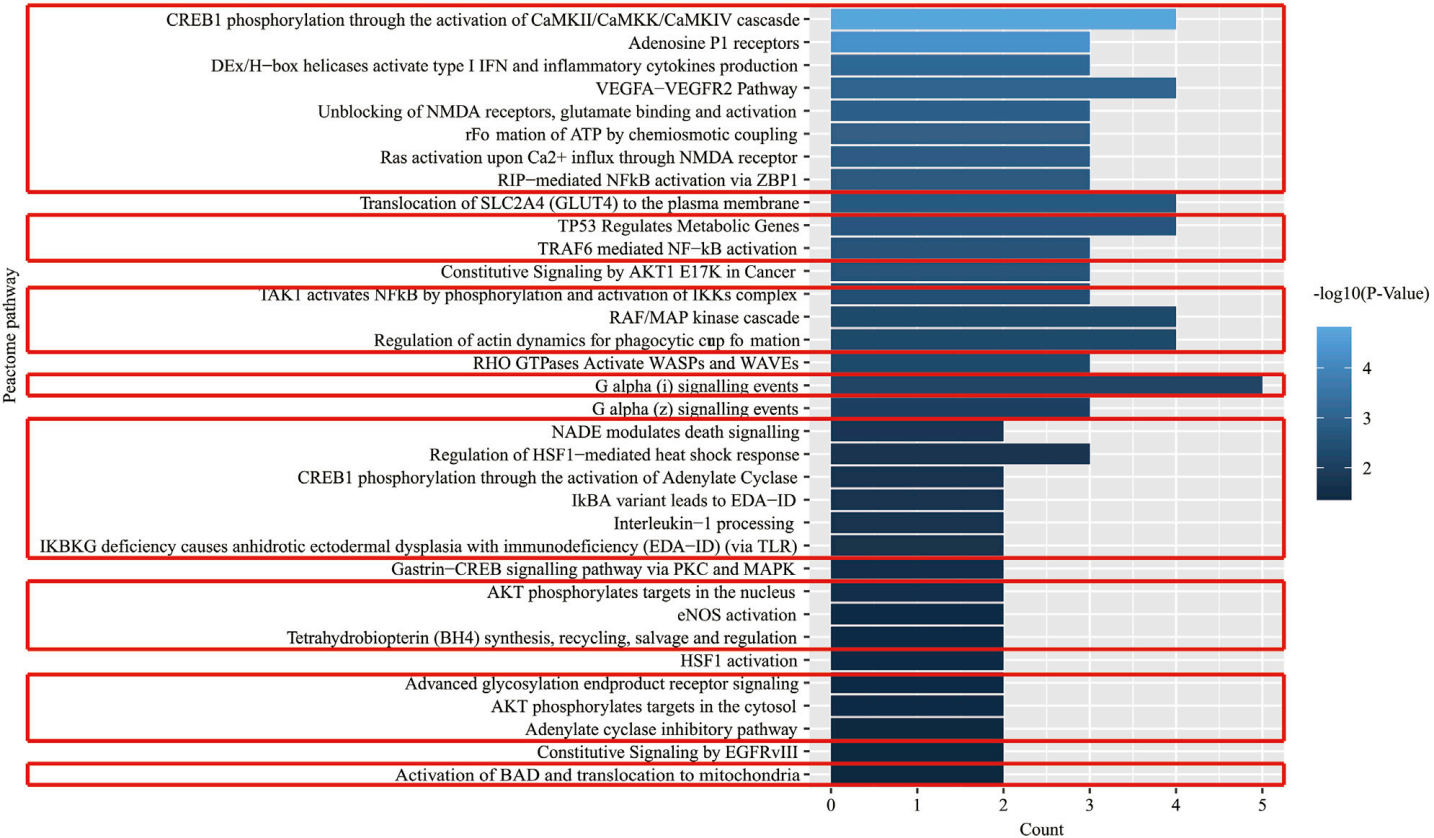
Pathway enrichment analysis of key effect genes of XLCQ in the treatment of stroke complicated with TRFS.

**FIGURE 7:**
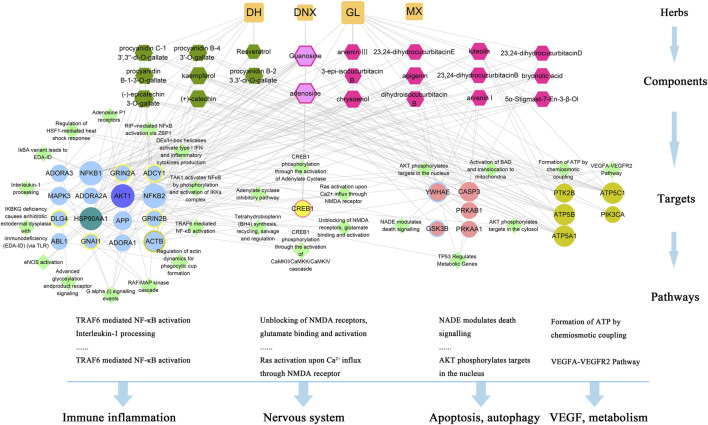
The network of interactions among 22 key active constituents of XLCQ, the corresponding 27 key targets and 27 pathways. [Square nodes refer to herbal medicine; hexagon nodes refer to key active constituents contained in three herbal medicines; circular nodes refer to putative targets (blue refers to inflammation-related, pink refers to apoptosis-related, yellows refer to nerve-related, brown refers to others, the color-overlay nodes refer to common genes of two sets, and purple or blue-green refers to three gene sets); and diamond nodes refer to pathways of putative gene enrichment].

**FIGURE 8:**
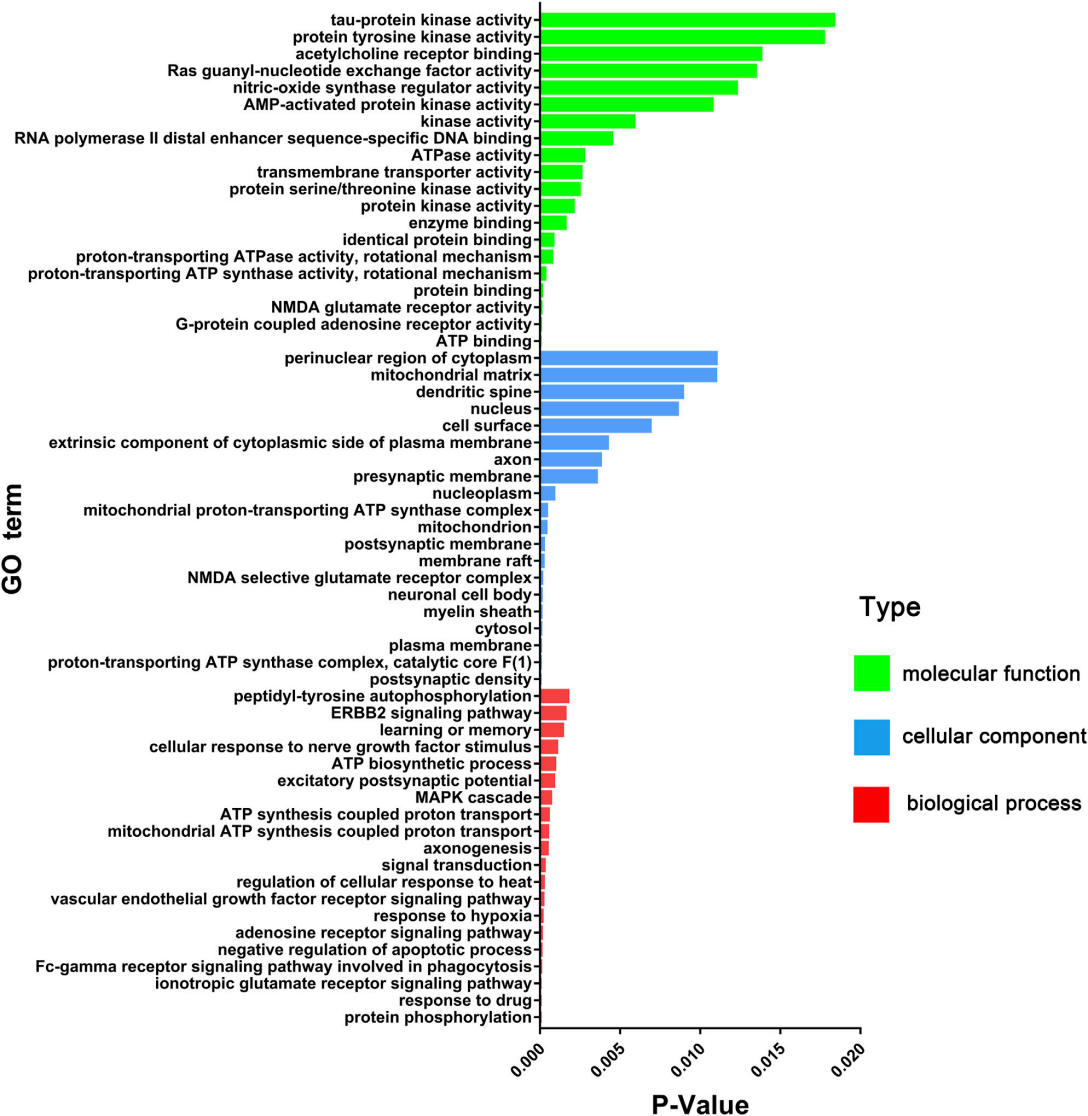
GO enrichment analysis (molecular function, cellular component, and biological process) of key effect genes of XLCQ in the treatment of stroke complicated with TRFS.

There were 60 chemical components corresponding to 150 major hubs. According to the number of major hubs and its frequency appearing in different pathways, the threshold was set at 2 and 13. That was, if a chemical constituent had more than two hubs and simultaneously the frequency of these hubs was more than 13, this chemical constituent was regarded as a key active constituent of XLCQ acting on stroke complicated with TRFS. Altogether, a total of 22 key active constituents and 28 corresponding targets were selected (Table 2). Twenty-seven of the 28 putative targets were enriched in 27 pathways (Figure 6, Supplementary Excel S3). The multi-dimensional network of 22 key active constituents, the corresponding 27 key targets, and 27 pathways was constructed as shown in (Figure 7). The GO functional analysis showed that these targets were mainly involved in immune inflammation (regulation of cellular response to heat, Fc-γ receptor signaling pathway involved in phagocytosis), the growth and development of the nervous system (ionotropic glutamate receptor signaling pathway, adenosine receptor signaling pathway, and cellular response to nerve growth factor stimulus), apoptosis (negative regulation of apoptotic process, MAPK cascade), and signal transduction (Figure 8).

**TABLE 2:** Active constituents of XLCQ and their putative targets.

NO.	Active constituents	Putative targets	Number of targets	Targets frequency	Category
1	**Guanosine** (−/24)^a^	MAPK3; DLG4; CREB1; AKT1; PIK3CA; ADORA3; ADORA2A; ADORA1; PRKAB1; PRKAA1; ADCY1; GNAI1; ABL1	13	42	*Trichosanthes kirilowii Maxim.Arisaema erubescens (Wall.) Schott*
2	**Adenosine** (50,20)	MAPK3; CREB1; AKT1; PIK3CA; ADORA3; ADORA2A; ADORA1; PRKAB1; PRKAA1; GSK3B; ADCY1; ABL1	12	39	*Trichosanthes kirilowii Maxim. Arisaema erubescens (Wall.) Schott*
3	5α-Stigmast-7-En-3-β-Ol (123/-)	GRIN2B; GRIN2A; NFKB1; NFKB2	4	24	*Trichosanthes kirilowii Maxim*
4	**Kaempferol** (160/103)	AKT1; HSP90AA1; ACTB; PTK2B; PRKCA; ATP5B; ATP5C1; ATP5A1	8	23	*Trichosanthes kirilowii Maxim*
5	Arvenin Ⅲ (169/111)	NFKB1; NFKB2; CASP3; YWHAE	4	19	*Trichosanthes kirilowii Maxim*
6	Arvenin I (186/126)	NFKB1; NFKB2; CASP3; YWHAE	4	19	*Trichosanthes kirilowii Maxim*
7	Dihydroisocucurbitacin B (−/140)	NFKB1; NFKB2; CASP3; YWHAE	4	19	*Trichosanthes kirilowii Maxim*
8	3-epi-isocucurbitacin B (209/138)	NFKB1; NFKB2; CASP3	3	17	*Trichosanthes kirilowii Maxim*
9	23,24-dihydrocucurbitacinD (−/124)	NFKB1; NFKB2; CASP3	3	17	*Trichosanthes kirilowii Maxim*
10	**Resveratrol** (114/61)	AKT1; APP	2	15	*Trichosanthes kirilowii Maxim*
11	**Procyanidin B-1-3-O-gallate** (118/64)	HSP90AA1; ACTB; PRKCA; ATP5B; ATP5C1; ATP5A1	6	13	*Trichosanthes kirilowii Maxim*
12	**Procyanidin B-2 3, 3′-di-O-gallate** (129/76)	HSP90AA1; ACTB; PRKCA; ATP5B; ATP5C1; ATP5A1	6	13	*Trichosanthes kirilowii Maxim*
13	**Procyanidin B-4 3′-O-gallate** (140/84)	HSP90AA1; ACTB; PRKCA; ATP5B; ATP5C1; ATP5A1	6	13	*Trichosanthes kirilowii Maxim*
14	**Bryonolic acid** (234/−)	GRIN2B; GRIN2A; NFKB1; NFKB2	4	24	*Rheum palmatum L*
15	**Luteolin** (162/−)	AKT1; HSP90AA1; ACTB; PTK2B; PRKCA; ATP5B; ATP5C1; ATP5A1	8	23	*Rheum palmatum L*
16	**Apigenin** (167/−)	AKT1; HSP90AA1; ACTB; PTK2B; ATP5B; ATP5C1; ATP5A1	7	20	*Rheum palmatum L*
17	Chrysoeriol (184/121)	AKT1; HSP90AA1; ACTB; PTK2B; ATP5B; ATP5C1; ATP5A1	7	20	*Rheum palmatum L*
18	**(−)-epicatechin 3-O-gallate** (132/79)	AKT1; HSP90AA1; ACTB; PTK2B; ATP5B; ATP5C1; ATP5A1	7	20	*Rheum palmatum L*
19	**(+)-catechin** (−/38)	AKT1; HSP90AA1; ACTB; PTK2B; ATP5B; ATP5C1; ATP5A1	7	20	*Rheum palmatum L*
20	23,24-dihydrocucurbitacin B (215/139)	NFKB1; NFKB2; CASP3; YWHAE	4	19	*Rheum palmatum L*
21	23,24-dihydrocucurbitacin E (-/128)	NFKB1; NFKB2; CASP3; YWHAE	4	19	*Rheum palmatum L*
22	**Procyanidin C-1 3′,** **3**″**-di-O-gallate** (128/75)	HSP90AA1; ACTB; PRKCA; ATP5B; ATP5C1; ATP5A1	6	13	*Rheum palmatum L*

a The number is corresponding to Supplementary Tables S1, S2.

### Luteolin, Apigenin, and Chrysoeriol Reduce the Release of IL-1β and TNF-α in LPS-Stimulated BV-2 Cells

**FIGURE 9:**
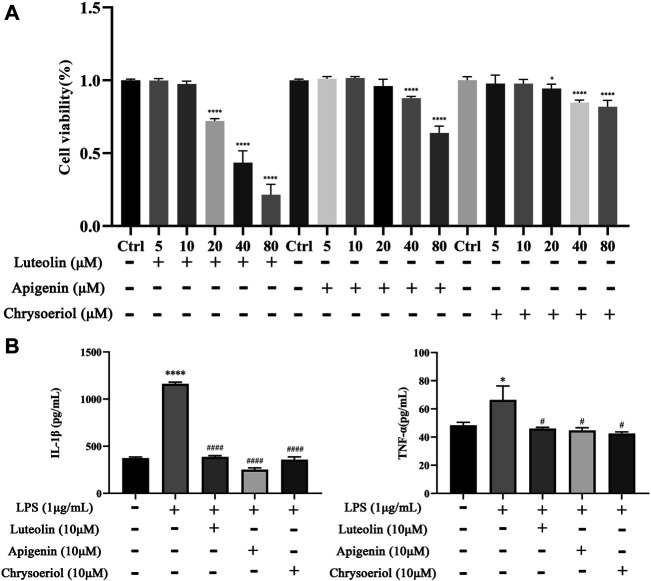
Luteolin, apigenin, and chrysoeriol prevents the neuroinflammatory response by LPS in BV-2 cells. Cytotoxicity was detected by the CCK-8 assay **(A)**. IL-1β and TNF-α levels were detected by ELISA kits **(B)**. (Mean ± SD, *p* < 0.05*^/#^, *p* < 0.0001****^/####^).

The CCK-8 assay kit was employed to evaluate the cytotoxicity to select the appropriate concentration of luteolin, apigenin, and chrysoeriol. As shown in Figure 9A, apigenin showed cytotoxicity to BV-2 cells at 40 μM, and luteolin and chrysoeriol showed cytotoxicity at 20 μM, so 10 μM was selected for a later anti-inflammatory study. Pro-inflammatory cytokine levels of IL-1β (*p* < 0.0001) and TNF-α (*p* < 0.05) were significantly increased after BV-2 cells were stimulated by LPS (1 μg/ml) for 24 h, and reversed by pretreatment with luteolin, apigenin, and chrysoeriol at 10 μM for 1 h, suggesting these three compounds had anti-neuroinflammatory activities (Figure 9B).

### Luteolin, Apigenin, and Chrysoeriol Downregulate the mRNA Expression of IL-1β, TNF-α, PIK3CA, AKT1, NFKB1, NFKB2, CREB1, and HSP0AA1

**FIGURE 10:**
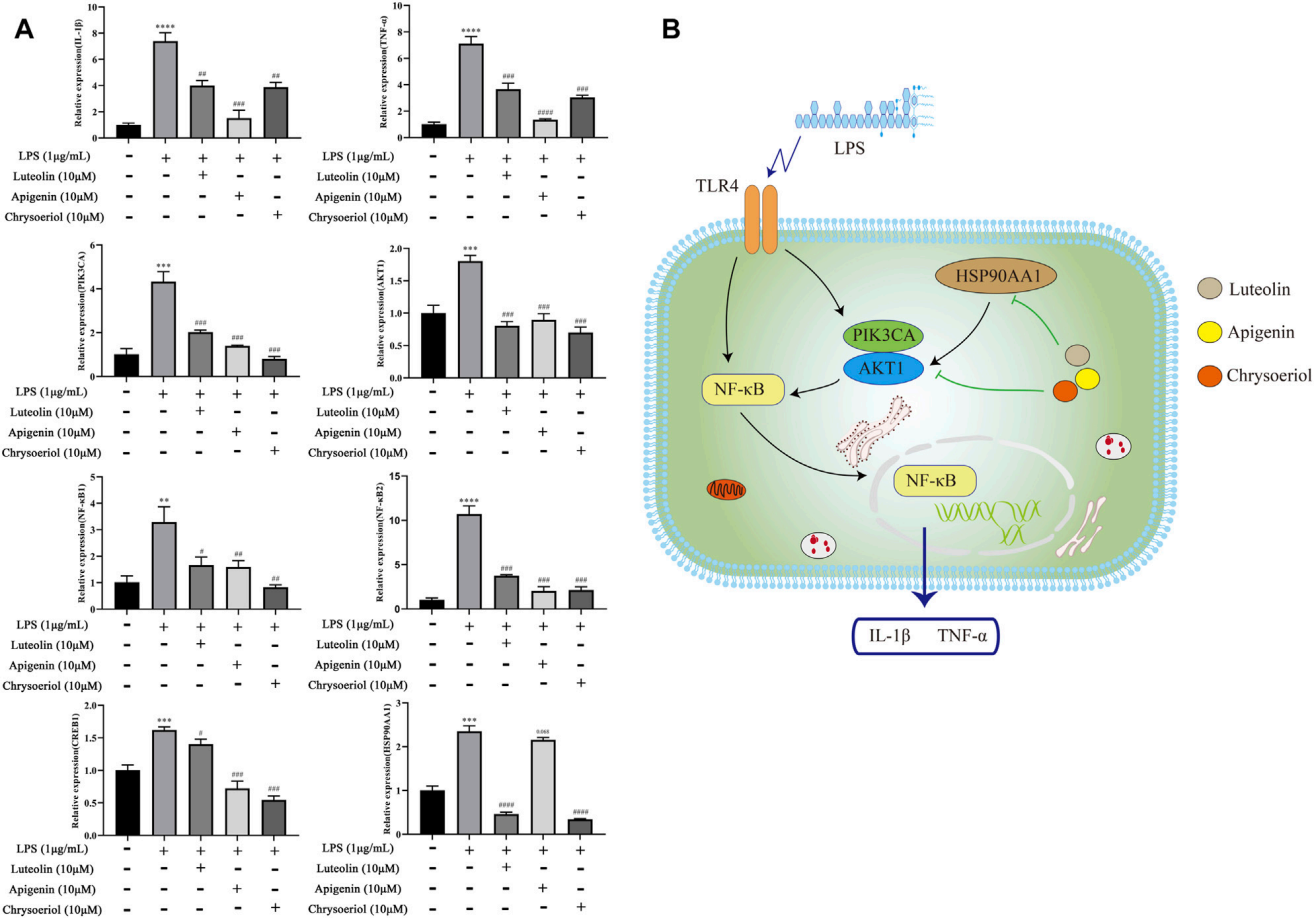
Luteolin, apigenin, and chrysoeriol downregulate the mRNA expression of IL-1β, TNF-α, PIK3CA, AKT1, NFKB1, NFKB2, CREB1, and HSP0AA1 **(A)**, and the pathway diagram is shown in **(B)**.

As shown in Figure 10A, the mRNA expression of IL-1β, TNF-α, PIK3CA, AKT1, NFKB1, and NFKB2 was 7.4-fold, 7.1-fold, 4.3-fold, 1.5-fold, 3.2-fold, 10.7-fold, 1.6-fold, and 2.3-fold increases, respectively, after the LPS stimulation, while pretreatment with these three compounds significantly reversed the above states. The pathway diagram is shown in Figure 10B.

## Discussion

In the present study, 197 chemical compounds were identified or tentatively characterized in the water extraction of XLCQ analyzed by UPLC-QTOF-MS/MS, and 176 of them had putative targets (Tanimoto score ≥0.8). *Natrii sulfas* had no putative targets as sodium sulfate was hydrolyzed to produce sulfate ion, which was not easily absorbed by the intestinal wall. *Natrii sulfas* remains in the intestine as a hypertonic solution to prevent the absorption of water in the intestine and increases the intestinal volume, thus causing mechanical stimulation and promoting the secretion of the intestinal wall. Subsequently, the heat pathogens and toxins in the body are discharged from the body. 833 putative targets corresponding to 176 components were predicted (Tanimoto Score ≥0.8) using TCMIP, and 159 stroke-related genes and 709 TRFS-related genes were collected from TCMIP. Twenty-two key active constituents were selected based on the interactions among the three abovementioned gene sets, which may improve the pathological state of stroke patients with TRFS syndrome by regulating the 27 corresponding targets that mainly involved in inflammation–immune-related pathways. Luteolin, apigenin, and chrysoeriol, as the predicted components, exhibited good anti-neuroinflammatory effects based on LPS-stimulated BV-2 cells.

“Integrative pharmacology” is an interdisciplinary science that comprehensively explores the interactions between the multiple constituents of TCM and the body at multiple levels (Xu et al., 2021). TCMIP, as an important part of integrative pharmacology, is an intelligent data mining platform that integrates big data management and pharmacology computing services, which consists of five databases and seven functional modules. The five database resources come from the encyclopedia of TCM (ETCM) (Xu et al., 2019), an international authoritative database, which provides the basic information of TCM, including chemical compounds, putative targets, corresponding disease, and syndrome. The seven functional modules can be used to query and mine the biological basis and mechanisms of disease, syndrome, and TCM.

Among the 22 constituents, ten of them have been reported to exert some protective effect on experimental cerebral ischemia (Table 2, marked in red), and their mechanisms were involved in several stroke-related targets and pathways, which was consistent with our corresponding targets and pathways. Guanosine, adenosine, luteolin, and kaempferol were reported to play a protective role in ischemic stroke by reducing neuroinflammation, oxidative stress, and excitotoxicity (Zhao et al., 2011; Bettio et al., 2016; Luo et al., 2019; Wang et al., 2020b). Apigenin protects from cerebral ischemia by reducing apoptosis and autophagy to promote cell regeneration through the Caveolin-1/VEGF pathway (Zachary, 2005; Pang et al., 2018; Cárdenas-Rivera et al., 2019). (−)-Epicatechin 3-O-gallate alleviated ischemia-reperfusion injury by promoting cell proliferation, angiogenesis, and migration, and inhibiting cell apoptosis and autophagy (Fu et al., 2019). (+)-Catechin could inhibit inflammatory biomarkers or cytokines, such as C-reactive protein, Lp-PLA2, IL-6, and TNF-α, to reduce ischemic injury (Tu et al., 2018). Resveratrol, a multifunctional biological polyphenol, was regarded as a potential drug for stroke-related diseases, which could alleviate hemorrhagic brain injury by inhibiting neuronal apoptosis (Zhao et al., 2019). It was reported that bryonolic acid could inhibit Ca^2+^ influx and regulate the gene expression in the Ca^2+^-CaMKII-CREB signaling pathway against cerebral ischemia (Que et al., 2016). Finally, procyanidins exert antioxidant activity against traumatic brain injury (Mao et al., 2015). The mechanisms of these ten chemical components covered most aspects of the pathological process of stroke, and the target and pathway information involved were consistent with our prediction results (Table 2; Figure 6), suggesting an integrative pharmacology strategy has certain prediction accuracy.

AKT1 and *PIK3CA* had high frequency in our study, and were involved in several signaling pathways, including TP53-regulated metabolic genes, AKT phosphorylates targets in the nucleus, AKT phosphorylates targets in the cytosol, activation of BAD and translocation to the mitochondria, eNOS activation, tetrahydrobiopterin (BH4) synthesis, recycling, salvage and regulation, and VEGFA-VEGFR2 pathway, which associated with apoptosis, inflammation, and nerves, and basically covers all mechanisms of stroke/TRFS, suggesting PI3K/Akt signaling pathways may play an important role in stroke formation (Yu et al., 2016; Lv et al., 2017; Pompura and Dominguez-Villar, 2018; Wen et al., 2018). PIK3CA was the only common target of key constituents and candidate stroke/TRFS targets, which may play an extremely important role in the treatment of XLCQ acting on stroke complicated with TRFS and needed to be focused on in the further experiment. MAPK3 was involved in encoding the proteins of the MAP kinase family. NFKB1 and NFKB2 were involved in encoding the NF-κB protein complex, which is an important nuclear transcription factor in cells, participating inflammatory response, immune response, apoptosis, stress response, *etc.* (Oeckinghaus and Ghosh, 2009; Mitchell et al., 2016). GSK3B was involved in the inflammation-related pathway regulation of HSF1-mediated heat shock response and apoptosis-related pathway AKT 101ylates targets in the cytosol. ATP5C1, ATP5A1, and ATP5B were involved in encoding a subunit of mitochondrial ATP synthase (Neupane et al., 2019). ADORA3, ADORA2A, and ADORA1, as adenosine receptors, were involved in inflammatory response, neuroprotection, apoptosis, and other intracellular signaling pathways (Chen et al., 2006; Blackburn et al., 2009; Sebastião and Ribeiro, 2009; González-Fernández et al., 2014; Feliu et al., 2019). CASP3 and HSP90AA1 were involved in the process of apoptosis and inflammation (Triantafilou et al., 2001; Creagh et al., 2003; Khurana and Bhattacharyya, 2015; Man and Kanneganti, 2016).

According to the prediction results, the rest 9 of the 22 constituents may be involved in anti-inflammatory, regulating energy metabolism, and antiapoptotic by regulating NFKB1, NFKB2, ATP5C1, ATP5A1, AKT1, HSP90AA1, *etc.* (Table. 2). We select three compounds (luteolin, apigenin, and chrysoeriol) with high response values (Supplementary Tables S1, S2) to perform some verification experiments. Luteolin and apigenin have been reported to restore ischemic brain injury of rodents (Ha et al., 2008; Luo et al., 2019). Chrysoeriol has been reported to reverse skin inflammation (Wu et al., 2020), arthritis (Ananth et al., 2016), and macrophage inflammation (Yoon and Park, 2021), except for neuroinflammatory effects. Inflammatory cascade is one of the major characteristics of stroke. Therefore, we evaluated their anti-neuroinflammatory effects based on LPS-stimulated BV-2 cells. TLR4 and PI3K/Akt signaling pathways were involved in regulating the activation of microglia and related cytokines in the process of neuroinflammation (Troutman et al., 2012; García et al., 2016; Rahimifard et al., 2017; Zhong et al., 2020). Excessive secretion of proinflammatory cytokines from BV-2 leads to a detrimental effect on neuronal cells (Wang et al., 2019). In our study, luteolin, apigenin, and chrysoeriol at 10 μM inhibited the release of IL-1β and TNF-α, as well as downregulated the mRNA expression of IL-1β, TNF-α, PIK3CA, AKT1, NFKB1, NFKB2, CREB1, and HSP0AA1. The TCM prescription emphasizes the synergistic effect of multi component, especially when treating complex diseases. An important research content of integrative pharmacology strategy is network prediction and verification. We delineate 22 key active constituents based on the integrative pharmacology strategy. Each component may only work for a certain link, and functional superposition of multiple components plays an overall effect. We selected three components with a high content for verification, which, of course, do not represent the efficacy of the whole prescription or directly reduce the infarct volume. Similarly, these three compounds are common in other botanical drugs and may have other effects.

In the present study, we just did some basic research about XLCQ, but the particular interactions between the active constituents and the corresponding targets, and if the 22 compounds could represent the whole prescription, still need to be verified in the near future.

## Conclusion

It is the first time to systematically analyze the chemical composition of XLCQ and to explore the pharmacological mechanisms of XLCQ acting on stroke complicated with TRFS using an integrative pharmacology strategy. The twenty-two key active constituents may improve the pathological state by regulating 27 corresponding targets that are mainly involved in inflammation–immune-related pathways. The integrative pharmacology strategy may offer a highly efficient way to mine the scientific connotation of traditional Chinese medicine prescriptions. This study might be a supplement for the deficiency of the basic research of XLCQ.

## Data Availability

The original contributions presented in the study are included in the article/Supplementary Material; further inquiries can be directed to the corresponding author.
